# Distribution of microsporidia in preterm and full-term infant gut microbiomes and implications for host health

**DOI:** 10.3389/fped.2025.1651866

**Published:** 2025-09-02

**Authors:** Anujit Sarkar, Maureen Groer, Thao T. B. Ho, Larry J. Dishaw

**Affiliations:** ^1^ College of Nursing, The University of Tennessee, Knoxville, TN, United States; ^2^ Department of Pediatrics, Division of Neonatology, Morsani College of Medicine, University of South Florida, Tampa, FL, United States

**Keywords:** ELBW infants, gut microbiome, metagenomics, microsporidia, VLBW infants

## Abstract

**Background:**

Microsporidia are a group of single-celled fungi which infect various chordates including humans, where they mainly pose a risk to immunocompromised individuals. This study aimed to investigate the occurrence of microsporidia in groups of very low birth weight (VLBW) and extremely low birth weight (ELBW) infants, comparing the findings with a publicly available dataset of term infant samples.

**Methods:**

Metagenomic sequencing was conducted on stool samples from two cohorts of preterm infants: cohort 1, which included 10 samples collected at 2, 4, and 8 weeks, and cohort 2, which consisted of 12 samples taken at 6 weeks and 2 years. These results were compared with data from a previously published cohort of term infants (cohort 3), which had 19 samples (in duplicates) collected between 1 and 14 weeks. Microsporidia identified from the data were separated and principal component analysis (PCA) was utilized to compare the microbiome of term and preterm infants. Microsporidia species that were significantly different between the two groups were identified using ALDEx2.

**Results:**

Early-stage microsporidia distribution did not show significant differences between the cohorts. However, significant differences emerged as the preterm infants grew, particularly at the age of 2 years (cohort 2). The levels of *Mitosporidium daphniae* (*p* = 0.03) and *Nematocida homosporus* (*p* = 0.04) were significantly higher in preterm infants compared to those born at term. Additionally, *Encephalitozoon romaleae* and *Nosema ceranae*, revealed an increase in cohort 2 from 6 weeks to 2 years.

**Conclusions:**

This manuscript reports, to the best of our knowledge, the first occurrence of microsporidia in the early stages of human life. Some microsporidia not only persist into childhood but also become more prevalent during this time. However, we wish to emphasize that the findings from this study should be interpreted with caution, considering the low sample size and comparing cohorts examined at different time points of infants' age. Future studies with larger sample sizes and more mechanistic approaches could help clarify their role in childhood development and long-term health.

## 1 Introduction

Microsporidia are predominantly intracellular single-celled fungi that lead intracellular lifestyles (1). Once classified as protists, microsporidia comprise about 220 genera and 1,700 species and possess highly reduced genomes, lack mitochondria, and have a polar tube that facilitates the infection of host cells; this infection creates a highly specific and obligate relationship with their eukaryotic hosts (2). Almost all animals, both vertebrates and invertebrates, can be infected by microsporidia. As opportunistic microorganisms, microsporidia thrive in immunocompromised individuals; however, an increasing number of species are recognized as emerging pathogens that persist in immunocompetent individuals (3, 4).

Currently, nine genera of microsporidia, *Anncaliia, Encephalitozoon, Enterocytozoon, Microsporidium, Pleistophora, Nosema, Trachipleistophora, Tubulinosema, and Vittaforma*, are known to infect humans. Among these, *Enterocytozoon* and *Encephalitozoon* are the most frequently reported in clinical settings (2, 3). They are considered opportunistic pathogens associated with diarrhea, malabsorption, and other systemic diseases and, historically, in persons suffering from AIDS (5). Disseminated microsporidia infection can be associated with high mortality in primates (6). Microsporidia generally follow single, host-specific life cycles except for a few, which can live in multiple hosts (2).

Microsporidia spores can be found in nearly all environments, persisting in surface and municipal water supplies (2). And because the spores are abundant and can be horizontally and vertically transferred, the aim of the current study was to identify and characterize their prevalence in the early human life. This understanding may help identify risks of infection and provide early therapeutic and/or interventional opportunities. This study utilizes a metagenomic sequencing approach to investigate the presence and persistence of microsporidia among cohorts of preterm infants and a publicly available term infant cohort. Importantly, this study is the first to report mitosporidia colonizing humans, particularly, premature and VLBW infants.

## 2 Materials and methods

The study comprised of three cohorts, which are described in Table 1. Briefly, two in-house cohorts (1 and 2), comprising preterm and very low birth weight (VLBW) infants, were utilized, while the third cohort comprised 19 term infant samples, in duplicate, from a previously published dataset (7).

**Table 1:** Study samples.

Cohort	Description	Sample size	Age range
1	VLBW and ELBW infants (2016–21)	30	2–8 weeks
2	Preterm, VLBW, and ELBW infants (2012–13)	12	6 weeks and 2 years
3	Term infants (PRJNA789149)	38	1–14 weeks

### 2.1 Sample collection and sequencing

Cohort 1: Infants in this cohort were enrolled as part of a larger prospective observational study. Very Low Birth Weight (VLBW, 1,500–1,000 grams) infants from a single level 3 academic neonatal intensive care unit (NICU) from August 2016 to August 2021 were included in this study. This dataset included 5 VLBW and 5 Extremely Low Birth Weight (ELBW infants) infants sampled at three time points (2, 4 and 8 week). The University of South Florida Institutional Review Board approved this study. All study procedures were performed in accordance with the hospital guidelines and clinical research safety regulations. The inclusion criteria were birth weight <1,500 grams, without major intestinal or chromosomal anomalies, and parental written informed consent as described previously (8). As the time of stool collection was very close (2, 4 and 8 weeks), they were considered independent samples while comparing with other cohorts. Sequencing libraries were constructed using the Nextera DNA Library Prep kit (Illumina) as per manufacturer's instructions and were sequenced on the NextSeq 2,000 sequencing platform (Illumina) employing a 2 × 150 bp chemistry.

Cohort 2: The collection of samples has been described previously in detail (9). Briefly, from a total of 83 infants comprising preterm, VLBW, and ELBW infants, 12 infants were selected for this study with stool samples collected at six weeks of age in the neonatal intensive care unit (NICU) at Tampa General Hospital during May 2012—Dec 2013. The stool samples were collected from diapers and frozen at −80°C until processing. Additional stool samples were collected from the same 12 infants at two years of age. The MoBio PowerFecal DNA kit (Qiagen, Carlsbad, CA) was utilized to extract the DNA for metagenomic analyses. The metagenomic sequencing was performed in a similar manner as the cohort 1.

Cohort 3: This cohort comprised term infant metagenomic sequence data from a previously published study (7). The ages in this cohort ranged from 1 to 14 weeks, and the raw data (fastq files) were downloaded from the NCBI Sequence Read Archive.

### 2.2 Bioinformatic analyses

Fastq files from the three cohorts were analyzed using the same pipeline. The raw fastq files were visualized for quality using FastQC (10) and filtered accordingly with Trimmomatic (11). The host sequences were removed from the clean sequences using Bowtie2 with the human reference genome GRCh38 (12). Non-human sequences were assumed to be of microbial origin and classified using Kaiju (13), employing the NCBI RefSeq for bacteria (May 2023 version) in each sample separately. The classified sequences were then tabulated and merged to obtain the combined taxonomies. As the primary focus of this manuscript is on microsporidia, all hits corresponding to microsporidia from all samples were separated, and a new table was constructed using the distribution of microsporidia across the samples.

### 2.3 Statistical analyses

Since the term infant's data was taken from a previously published dataset, an appropriate batch effect correction could not be achieved to differentiate with the in-house data which was exclusively from preterm infants. However, PCA was utilized to visualize if the cohorts differ significantly due to batch effects. Since the sequencing depth varied with the cohort, the microsporidia counts were adjusted employing the Trimmed mean of M values (TMM) for further comparisons. To compare if the abundances of microsporidia species differed when the infants grew from 6 weeks to 2 years in cohort 2, paired Wilcoxon signed-rank test was utilized. A phyloseq object was created based on the distribution of microsporidia and comparison within the groups were performed within the phyloseq object in R.

Comparison between terms and preterm infants with microsporidia colonization based upon week of sample collection were performed employing PCA. The microsporidia species significantly different between the preterm and term infants were identified using ALDEx2 (14).

## 3 Results

**Figure 1:**
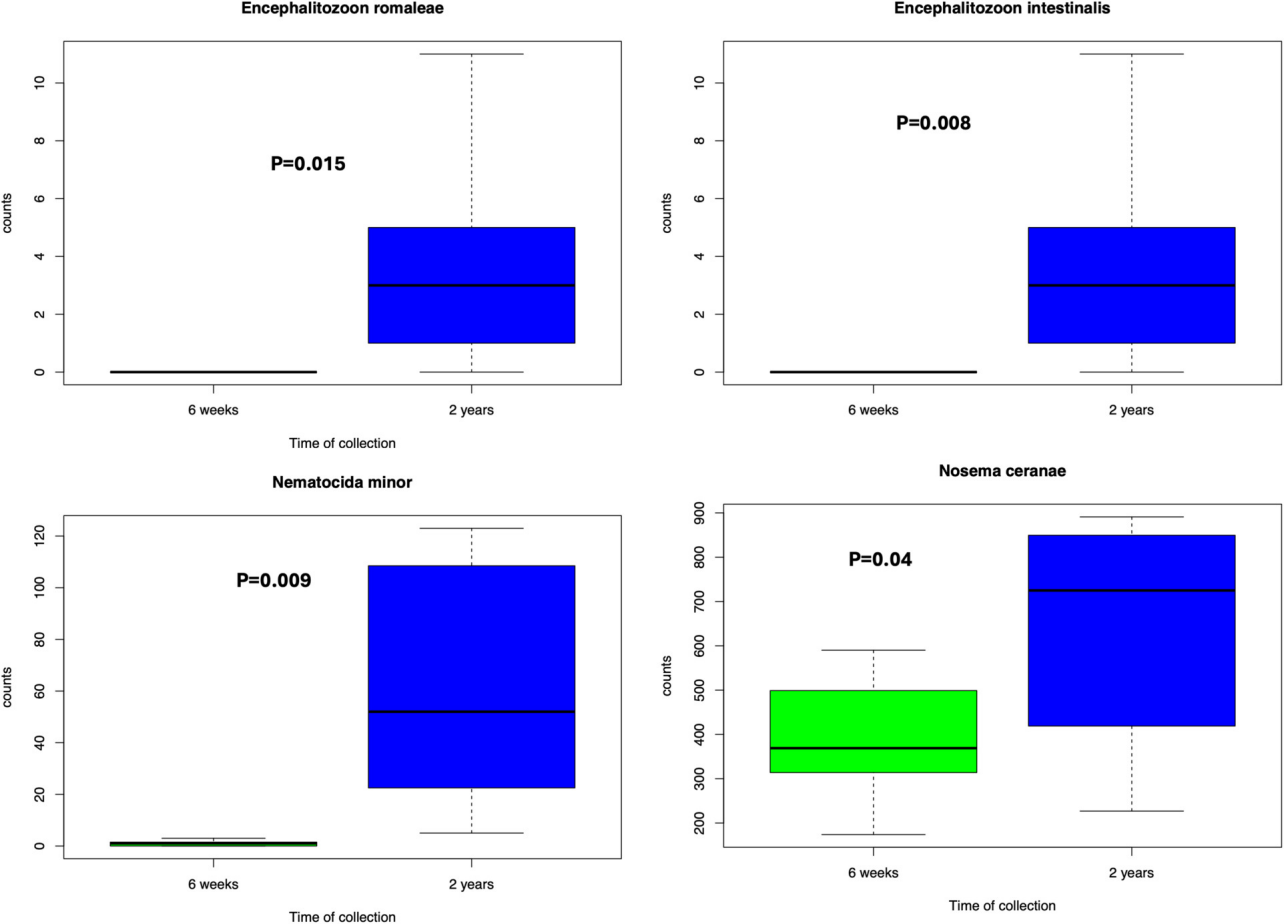
Microsporidia significantly more abundant at 2 years compared to 6 weeks from cohort 2. Wilcoxon test was performed based on the abundances and a *p*-value less than 0.05 was considered significant.

**Figure 2:**
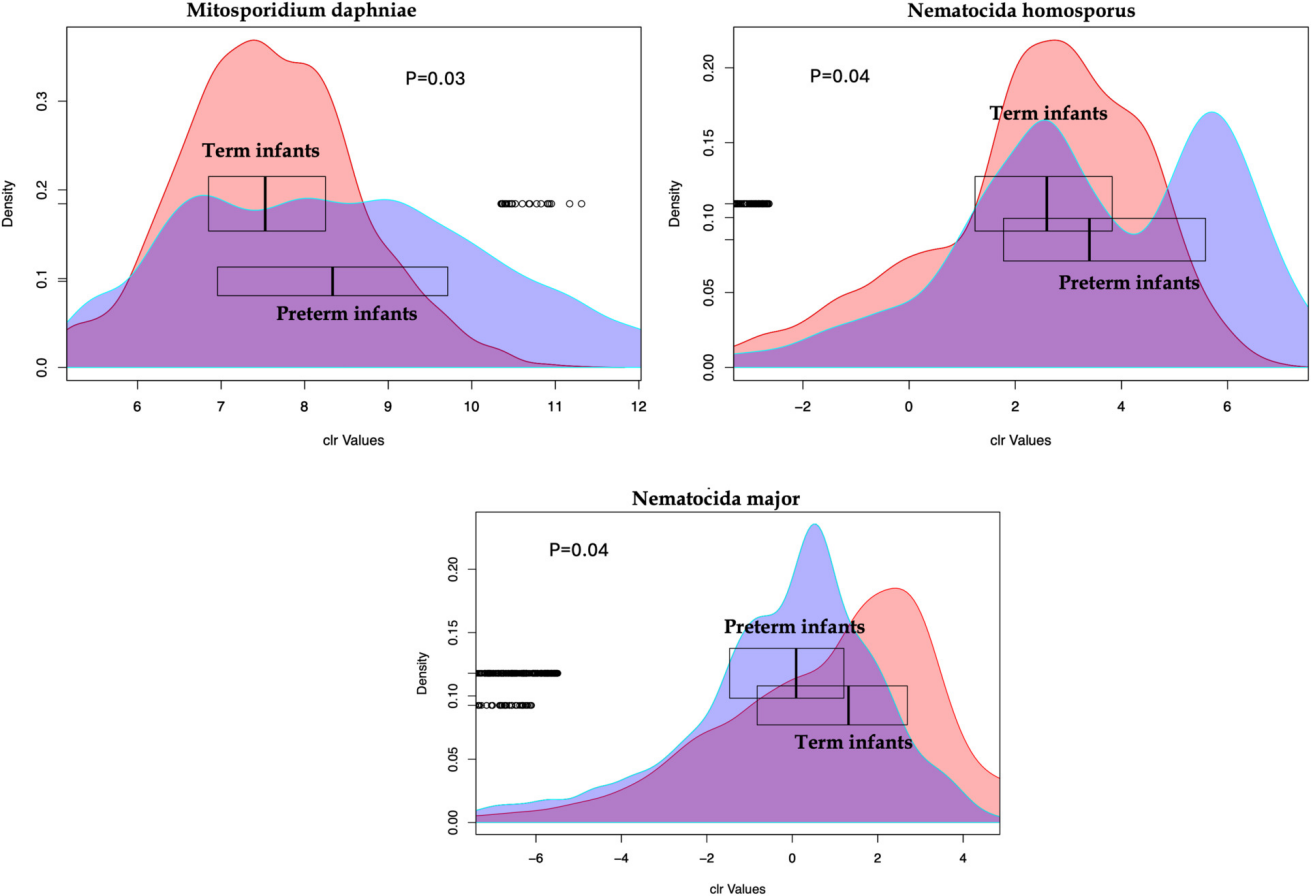
Microsporidia species with abundances significantly different between the preterm and the term infants. The *X*-axis shows the centered log ratio (clr) transformed values of abundances while the *Y*-axis show the distribution of the values. The box plots for each group show the median for each group.

In comparing preterm and term infant cohorts, mitosporidium genus was found to be more abundant in the preterm cohorts (Supplementary Figure S1). PCA analyses revealed no significant clustering based on the batches, indicating limited batch effects and justifying the combined analyses of all samples (Supplementary Figure S2). No significant differences were noted in the distribution of microsporidia species among the three cohorts while comparing the preterm infants against the terms (Supplementary Figure S3). However, the distribution of microsporidia did differ between early life (week stage) and at two years of age. The PCA plot (Supplementary Figure S4) reveals a distinct distribution of microsporidia among children at 2 years of age compared to the preterm samples or compared to infants born at term (cohort 3). Upon comparing the microsporidia of preterm infants and at two years for the same samples from cohort 2, a few observations remain. First, some of the microsporidia were significantly increased (Figure 1) at 2 years of age than at the preterm infant stage (6 weeks). And second, in comparing the preterm and term cohorts, several microsporidia were found to be significantly different between the groups (Figure 2).

## 4 Discussion

This study is the first, to our knowledge, to report the presence and distribution of various species of microsporidia during early human development. Cohorts one and two were collected from unrelated donors in the same neonatal intensive care unit (NICU) in the Tampa area, from two separate studies and 4–9 years apart. Cohort three includes samples from a previously published study conducted in an unrelated NICU, in a different city with different donors. Although it remains unclear whether the microsporidia are being transmitted horizontally or vertically, the distinct composition observed during prematurity—when infants are often underdeveloped and immunocompromised—deserves special attention. The dissemination of microsporidia in immunocompromised individuals can lead to severe consequences, such as malnourishment and growth restrictions, which can be fatal (see below). Infants born prematurely and sampled after 2 years seem to retain a unique microsporidia composition compared to infants born at term.

As obligate intracellular parasites, most microsporidia have reduced their genomes, and lost their mitochondria, instead depending on the host for ATP and energy production. Microsporidia are the only known eukaryotes to have lost nearly all the genes encoding the mechanistic target of rapamycin, or mTOR, which regulates cell division, energy harvest, and replication; the intracellular parasites instead deprive mTOR pathway components of its host cells (15). Thus, increased parasitic burden can lead to unprecedented risks for epithelial cell renewal and metabolic dysfunction in the intestines (1, 16, 17).

The spores of these parasites are uniquely characterized by having a polar tube or filament that is used to infiltrate host cells (1). While historically known to infect mostly arthropods and fish, these intracellular fungi have gained increased attention because of their additional ability to infect the intestinal epithelium of humans, thriving via dissemination in those that are immunocompromised, and recently recognized to be capable of broad-host range preferences (2, 16). Their infectious burden was originally described in one of the largest case studies among HIV patients (18–21) and remains a significant pathogen in most reported cases (2, 4, 22).

While vertical transmission has been observed in various animals, it has not yet been documented in humans (2). Yet, the ability to thrive in the food chain suggests that in addition to water, horizontal transfer of microsporidia is likely to occur in the food supply of humans (22). Of relevance to both immunocompetent and immunocompromised individuals, host immune factors regulating microsporidia infection have been shown to impact the susceptibility to bacterial pathogens in a non-human model system (23). Additionally, neurologic syndromes and microsporidiosis have been correlated following solid organ transplantation (24). Thus, it has been recommended that clinicians consider microsporidia burden (or infections) when encephalitis is suspected, even without gastrointestinal symptoms (2, 22, 24).

Microsporidia are known to cause significant diarrhea, wasting, and malabsorption (5), and among the immunocompromised, dissemination can be fatal (25). Increased burden, dissemination, and added complications associated with this intracellular pathogen should be considered in this vulnerable population of very and extremely low birth weight infants. With an emerging number of infections being reported in immunocompetent individuals (25, 26), compositional changes in microsporidia abundance should be considered in term infants that develop gastrointestinal symptoms that do not resolve. An important limitation of the current study is that 2-year samples were available only for VLBW infants and not for the term infant cohort. In addition, the sample size of our cohort is too low to reliably conclude on the abundances of microsporidia in these infant populations. Although the presence of microsporidia is generally not considered common in humans, this is not the first or only study to identify their presence at an early age. A previous study (27) reported the presence of microsporidia in preschool children. Based on this current study, it is difficult to ascertain if the microsporidia in premature infants were horizontally acquired from the NICU environment or vertically transferred during gestation or birth; however, to the best of our knowledge, this is the first report of persistent microsporidia identified in premature infants of disparate NICU environments. Follow-up investigations should examine if term infants retain certain microsporidia upon reaching two years of age, or if this finding is restricted to infants born VLBW.

## Data Availability

The raw data of the metagenomic sequencing generated in this study were submitted to the European Nucleotide Archive under project number PRJEB96252.

## Supplementary material

Supplementary Figure 1 Distribution of main genera across the three cohorts. The genus Mitosporidium was visibly more abundant in the preterm cohorts (A and B) than in the term infants (cohort C).

Supplementary Figure 2 PCA plot for the three cohorts considered as different batches. Batch A, B, and C represent cohorts 1, 2, and 3, respectively. No clear separation of the cohorts was observed, indicating no significant batch effects.

Supplementary Figure 3 PCA plot of preterm and term infants based on microsporidia distribution. No significant differences were observed between the two groups.

Supplementary Figure 4 PCA plot of all infants based on time of sample collection. The samples at two years from cohort 2 clustered separately indicating different microsporidia distribution from all other infants.
