# Supplementary figures and images for: Distribution of microsporidia in preterm and full-term infant gut microbiomes and implications for host health
Source: Front Pediatr. 2025 Sep 2;13:1651866. doi: 10.3389/fped.2025.1651866 (PMC12436282; doi:10.3389/fped.2025.1651866)

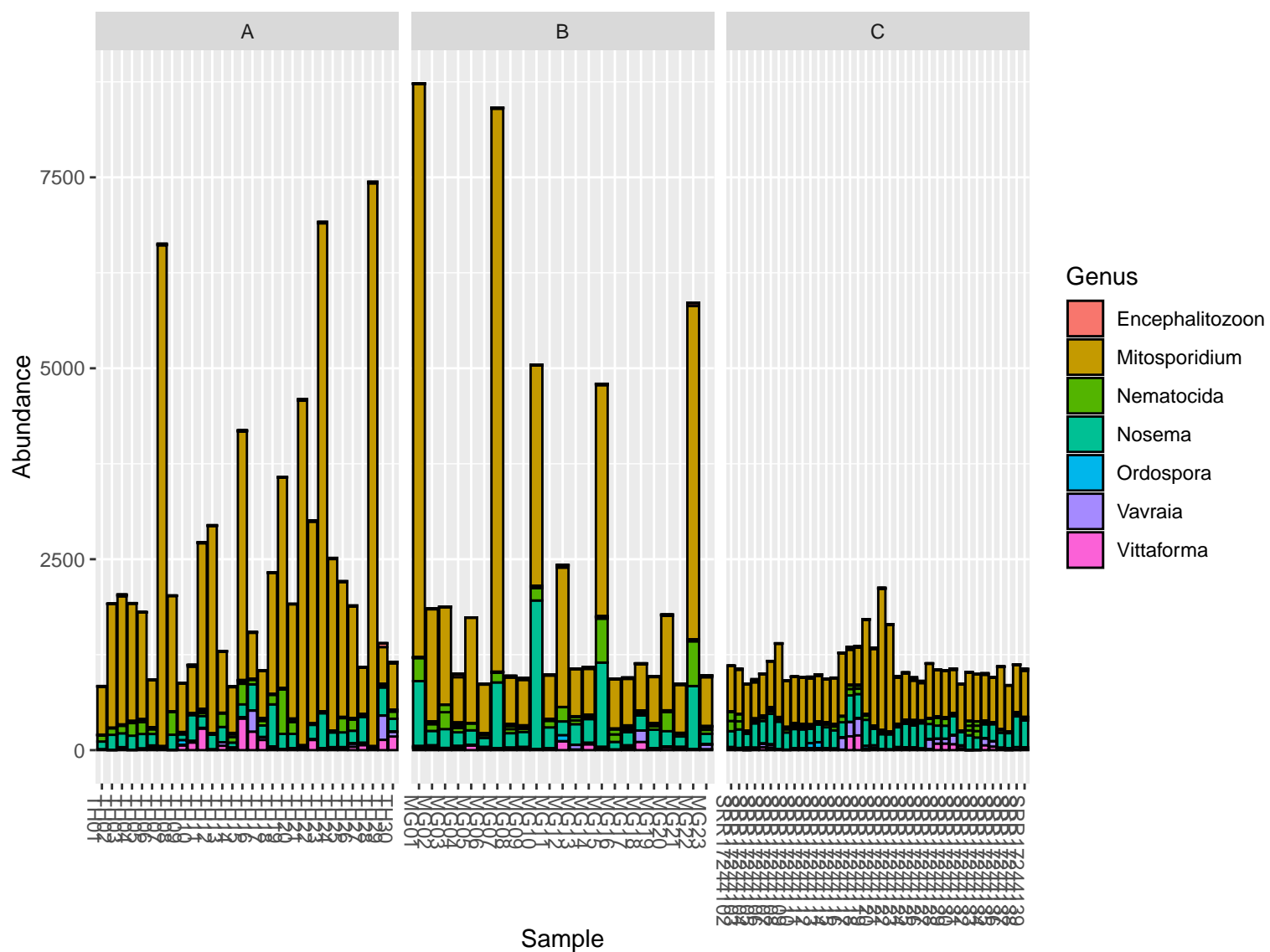

Supplement: Supplementary Figure 1 — Distribution of main genera across the three cohorts. The genus Mitosporidium was visibly more abundant in the preterm cohorts (A and B) than in the term infants (cohort C). [file Datasheet1.pdf]

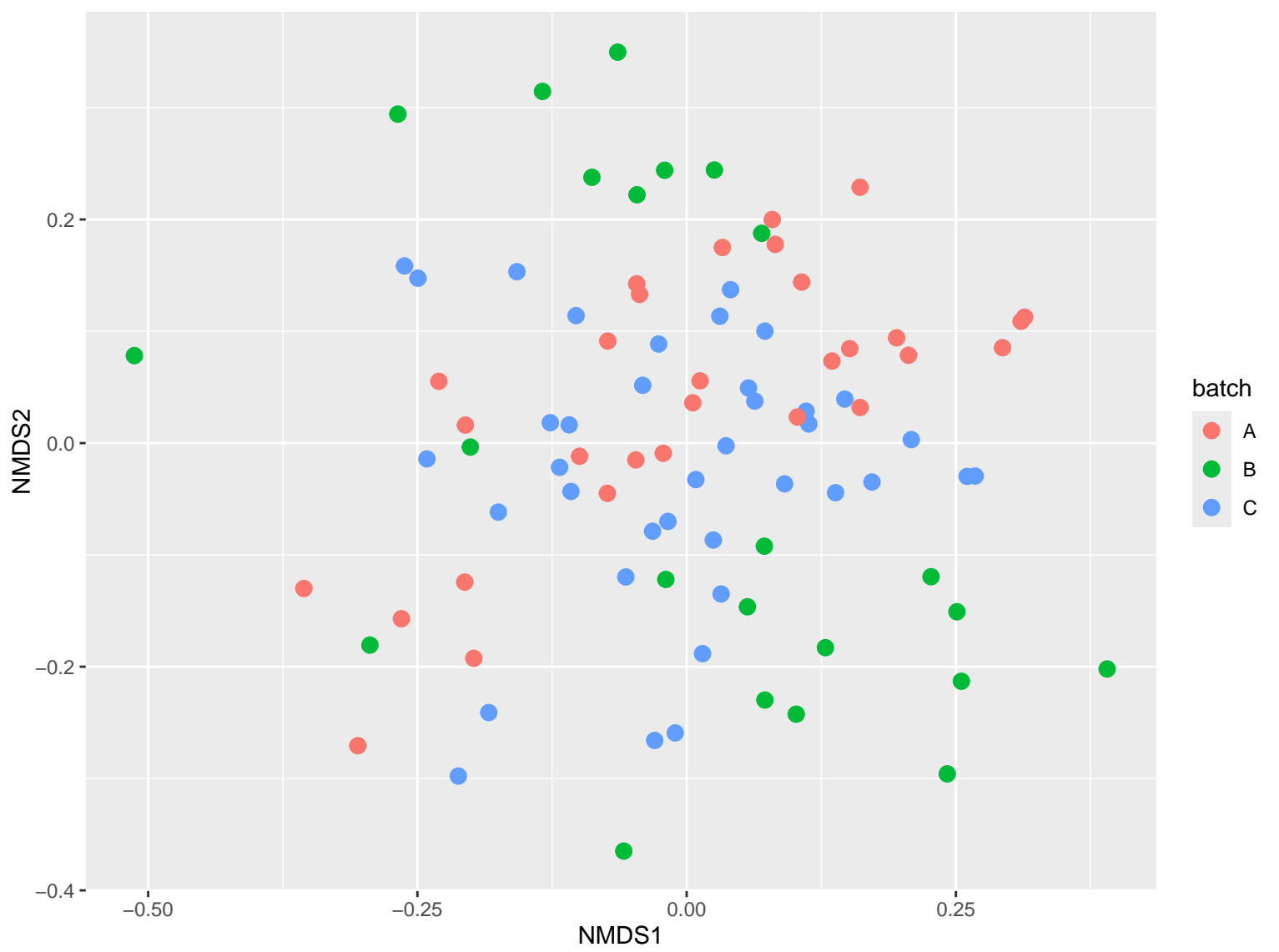

Supplement: Supplementary Figure 2 — PCA plot for the three cohorts considered as different batches. Batch A, B, and C represent cohorts 1, 2, and 3, respectively. No clear separation of the cohorts was observed, indicating no significant batch effects. [file Datasheet2.pdf]

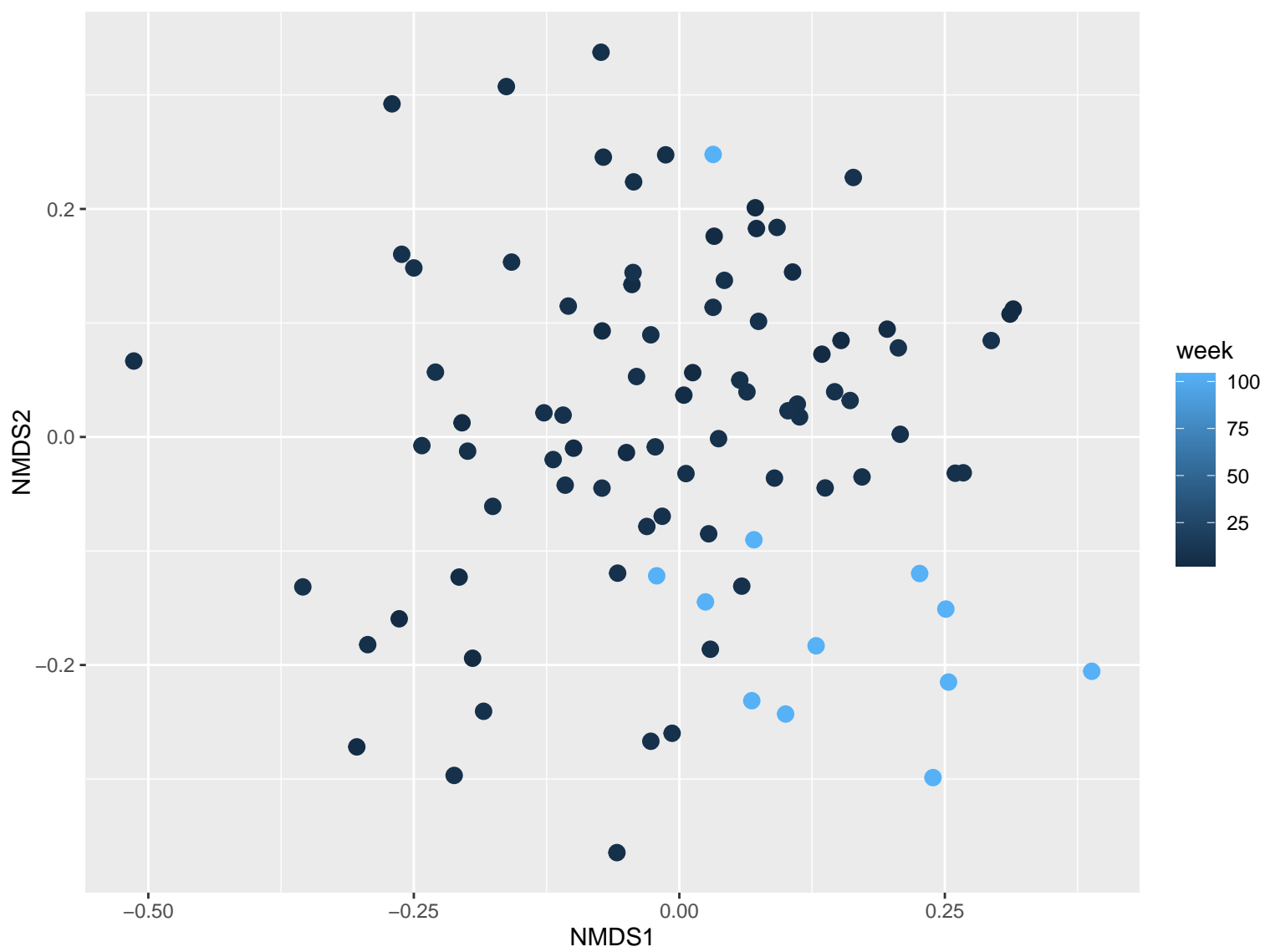

Supplement: Supplementary Figure 3 — PCA plot of preterm and term infants based on microsporidia distribution. No significant differences were observed between the two groups. [file Datasheet3.pdf]

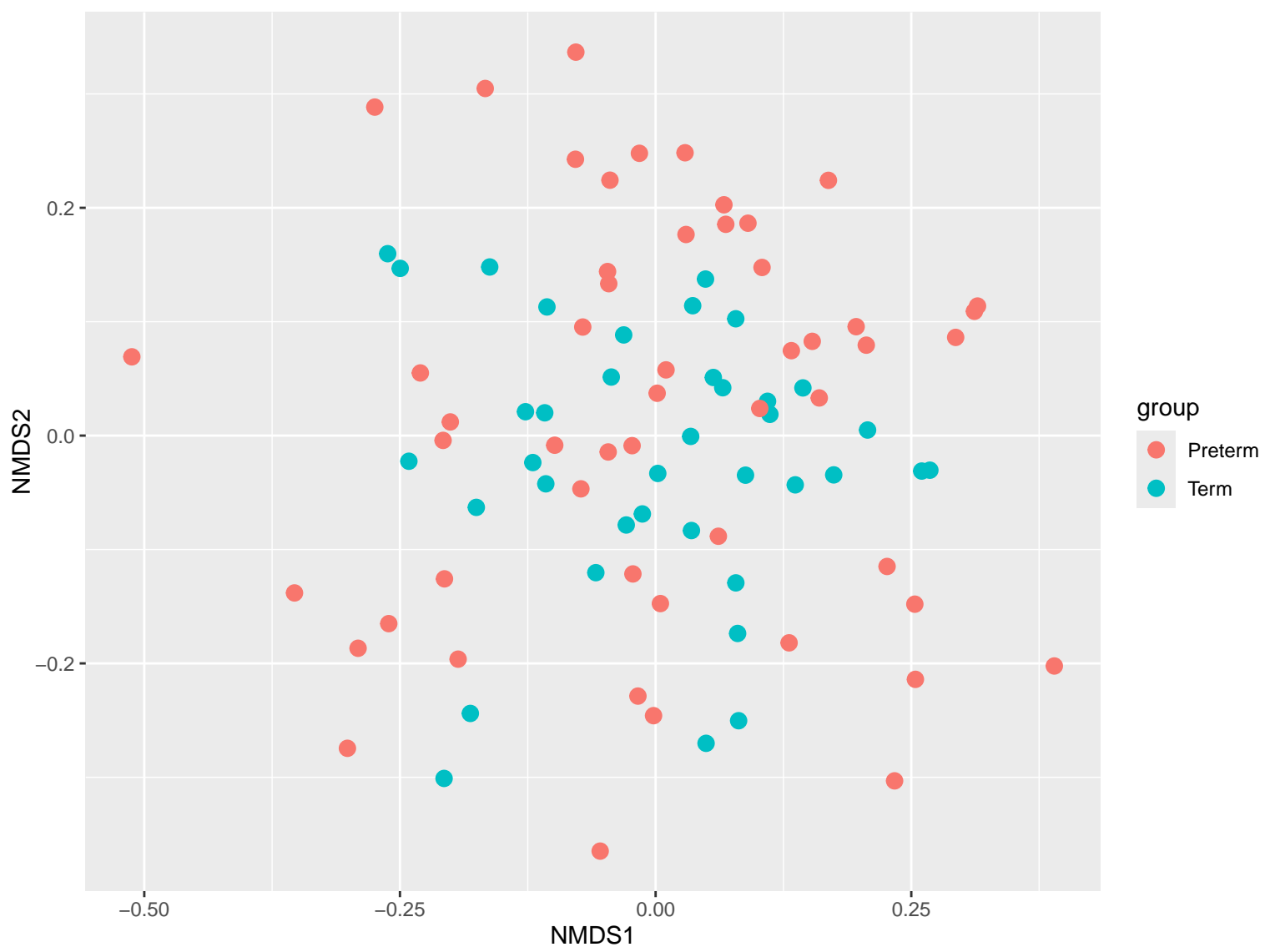

Supplement: Supplementary Figure 4 — PCA plot of all infants based on time of sample collection. The samples at two years from cohort 2 clustered separately indicating different microsporidia distribution from all other infants. [file Datasheet4.pdf]
